# Interferon-induced protein with tetratricopeptide repeats 3 may be a key factor in primary biliary cholangitis

**DOI:** 10.1038/s41598-021-91016-6

**Published:** 2021-06-01

**Authors:** Motoko Sasaki, Yasunori Sato, Yasuni Nakanuma

**Affiliations:** 1 Department of Human Pathology, Kanazawa University Graduate School of Medical Sciences, Kanazawa, 920-8640 Japan; 2 Department of Pathology, Fukui Saiseikai Hospital, Fukui, 918-8503 Japan

**Keywords:** Diseases, Gastroenterology, Pathogenesis

## Abstract

Accumulating studies suggest that senescent biliary epithelial cells (BECs) produce senescence-associated secretory phenotypes (SASPs) and play various roles in the pathogenesis of primary biliary cholangitis (PBC) and other cholangiopathies. We examined comprehensive profiles of senescent BECs and its contribution to the pathogenesis of PBC taking advantage of microarray analysis. cDNA microarray analysis revealed that 1841 genes including *CCL2*, *IFIT3, CPQ* were commonly up-regulated in senescent BECs cultured in serum depleted media or media with glycochenodeoxycholic acid*.* Knockdown of *IFIT3* significantly suppressed cellular senescence (*p* < 0.01) and significantly increased apoptosis (*p* < 0.01) in BECs treated with serum depletion or glycochenodeoxycholic acid. Significantly increased expression of IFIT3 was seen in senescent BECs in small bile ducts showing cholangitis and in ductular reactions in PBC, compared to control livers (*p* < 0.01). An inadequate response to UDCA was inversely correlated to the increased expression of IFIT3 in small bile duct in PBC (*p* < 0.05). In conclusion, the expression of various genes related to immunity and inflammation including SASPs were increased in senescent BECs. Upregulated IFIT3 in senescent BECs may be associated with the pathogenesis of PBC and may be a possible therapeutic target in PBC.

## Introduction

Primary biliary cholangitis (PBC) is an autoimmune cholestatic liver disease characterized by a unique chronic non-suppurative destructive cholangitis (CNSDC) in small bile ducts and serum anti-mitochondrial antibodies (AMAs)^1–3^. PBC presents progressive chronic cholestasis and biliary fibrosis, which subsequently develop liver failure. There still remains issues to be clarified in the pathogenesis of PBC, so far^1–3^. The only accepted first-line agent is ursodeoxycholic acid (UDCA) for the treatment of PBC^1–4^. Approximately one-third of patients treated with UDCA are “nonresponders” to UDCA at risk of disease progression prompting the need for additional therapeutic strategies^1–4^.

PBC is characterized by biliary epithelial senescence in small bile duct and bile ductules^5–12^. Definition of cellular senescence is a condition in which a cell no longer has the ability to proliferate^9,13,14^. Irreversible G1/S arrest of cell cycle is a feature of senescent cells^9,13,14^. Senescent cells do not respond to various external stimuli, although they remain metabolically active^9,13,14^. Cellular senescence is observed in the damaged small bile duct involved in CNSDC and also bile ductular cells in ductular reactions in our previous studies^5,6^. Although exact mechanisms inducing cellular senescence in PBC remains unknown, so far, cellular senescence can be induced by treatments with oxidative stress, serum depletion or glycochenodeoxycholic acid (GCDC) in cultured BECs in our previous studies^5,8,12,15^. It is conceivable that senescent BECs may participate in the pathogenesis of PBC and other various cholangiopathies by secreting senescence-associated secretory phenotypes (SASPs), such as augmented inflammation, progression of fibrosis and bile duct loss^8,9,13–16^.

Although accumulating data suggest important roles of senescent BECs in hepatobiliary diseases^5–12^, features of senescent BECs have not been comprehensively studied, so far. Taking advantage of cDNA microarray analysis, we examined RNA expression profiles of senescent BECs and its contribution to cholangiopathies. Results of cDNA microarray analysis showed that some of the commonly up-regulated genes in senescent BECs following in vitro incubation in serum depleted media or treatment with GCDC included *CCL2*, *IFIT3 CPQ*. We chose to focus on the interferon (IFN)-induced protein with tetratricopeptide repeats 3 (IFIT3), since it showed the greatest increase in levels of gene expression. IFIT3 is an IFN-induced antiviral protein acting as an inhibitor of cellular and viral processes, cell migration, proliferation, signaling, and viral replication^17–19^. A participation of IFN pathways in the pathogenesis of PBC was previously reported^20–24^, so we selected *IFIT3* for further examination.

In this study, we examined the effects of knockdown of IFIT3 on cellular senescence, proliferation and apoptosis in cultured BECs. We also examined the expression of IFIT3 and its association with senescent markers p16^INK4a^ and p21^WAF1/Cip1^ in human PBC and control livers.

## Results

### Culture study

#### A number of genes was upregulated in senescent BECs induced by serum depletion and GCDC treatment

Upregulated genes in senescent BECs, which show more than twofold change compared to control, were 2870 and 2789 genes in serum depletion and GCDC treatment for 7 days, respectively. 1841 genes were commonly upregulated in both 2 conditions. Major genes commonly upregulated in senescent BECs induced by serum depletion and GCDC treatment included *CCL2, IFIT3, CPQ, NUPR1 and CIB2* (Table 1, Supplementary table S1). Various inflammatory genes (chemokines and cytokines) including *CCL2, CCL20, IL3, IL11, IL15* were upregulated in senescent BECs as SASPs (data not shown) in accordance with our previous study^8,16^. We put focus on *IFIT3 and* further examined its role in cell proliferation, apoptosis and cellular senescence. GSEA was also performed on top 500 up-regulated genes derived from senescent BECs induced by serum depletion and GCDC treatment. Various gene-sets were upregulated in each condition and a gene set: GO_RESPONSE_TO_BIOTIC_STIMULUS was commonly upregulated in both condition and IFIT3 was included in several gene sets including this gene set (Supplementary Table S2).

**Table 1:** Genes upregulated in senescent biliary epithelial cells (BECs) induced by GCDC treatment ant serum depletion.

Gene	Description	Senescent, dep	Senescent, GCDC
*Ccl2*	Chemokine (C–C motif) ligand 2	24.73	10.12
*Ifit3*	Interferon-induced protein with tetratricopeptide repeats 3	42.50	12.08
*Cpq*	Carboxypeptidase Q	10.68	9.72
*Nupr1*	Nuclear protein transcription regulator 1	13.72	9.21
*Cib2*	Calcium and integrin binding family member 2	8.24	9.70
*Fhod3*	Formin homology 2 domain containing 3	12.56	10.01
*Dtna*	Dystrobrevin alpha	28.92	19.61
*Gstm5*	Glutathione S-transferase, mu 5	14.29	8.15
*2010300F17Rik*	RIKEN cDNA 2010300F17 gene	17.18	14.98
*Tceal8*	Transcription elongation factor A (SII)-like 8	24.76	25.27
*Pik3r6*	Phosphoinositide-3-kinase, regulatory subunit 6	24.32	8.55
*Itpka*	Inositol 1,4,5-trisphosphate 3-kinase A	48.11	11.74
*Pyy*	Peptide YY	27.12	11.51
*H2-Ab1*	Histocompatibility 2, class II antigen A, beta 1	11.30	25.98
*Akr1e1*	Aldo–keto reductase family 1, member E1	8.08	8.27
*Sprr2b*	Small proline-rich protein 2B	12.89	8.89
*Sp5*	Trans-acting transcription factor 5	32.18	8.26
*Igsf1*	Immunoglobulin superfamily, member 1	10.30	8.61

The fold difference was obtained by dividing the mRNA level in senescent BECs with that in control BECs.

*Dep* serum depletion, *GCDC* glycochenodeoxycholic acid (detail data are shown in Supplemental Table S2).

#### IFIT3 expression was increased in senescent cells in mRNA and protein level

**Figure 1:**
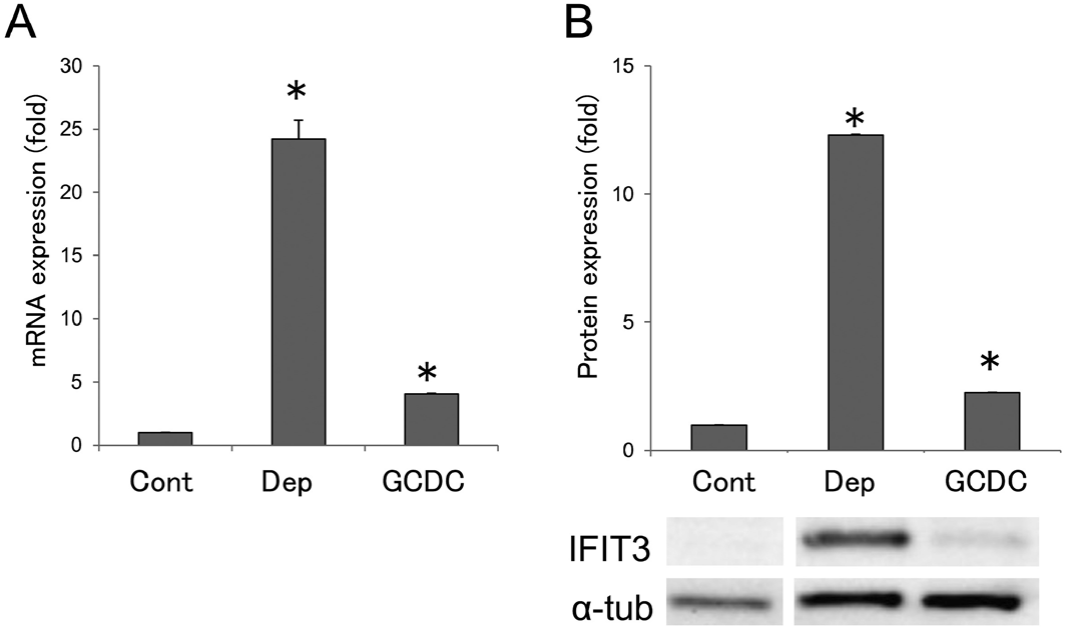
IFIT3 expression was increased in senescent cells in mRNA and protein level. (**A**) The expression of Ifit3 mRNA was significantly increased in senescent BECs induced by serum depletion and GCDC. *p < 0.01 compared to control. (**B**) The expression of IFIT3 protein was significantly increased in senescent BECs induced by serum depletion and GCDC. *p < 0.01 compared to control. Blots were cropped from different parts of the same gel/blot (Supplementary information 3).

We examined the mRNA expression of *IFIT3* in cultured BECs treated with serum depletion and GCDC for 7 days. The expression of *IFIT3* mRNA was significantly increased in senescent BECs treated with serum depletion and GCDC, compared with control (*p* < 0.01) (Fig. 1A). The expression of IFIT3 at protein level was also significantly increased in senescent BECs treated with serum depletion and GCDC, compared with control (*p* < 0.01) (Fig. 1B).

#### *IFIT3* knockdown increased cell proliferation, apoptosis and decreased cellular senescence in BECs treated with serum depletion and GCDC

##### Effective knockdown of IFIT3

**Figure 2:**
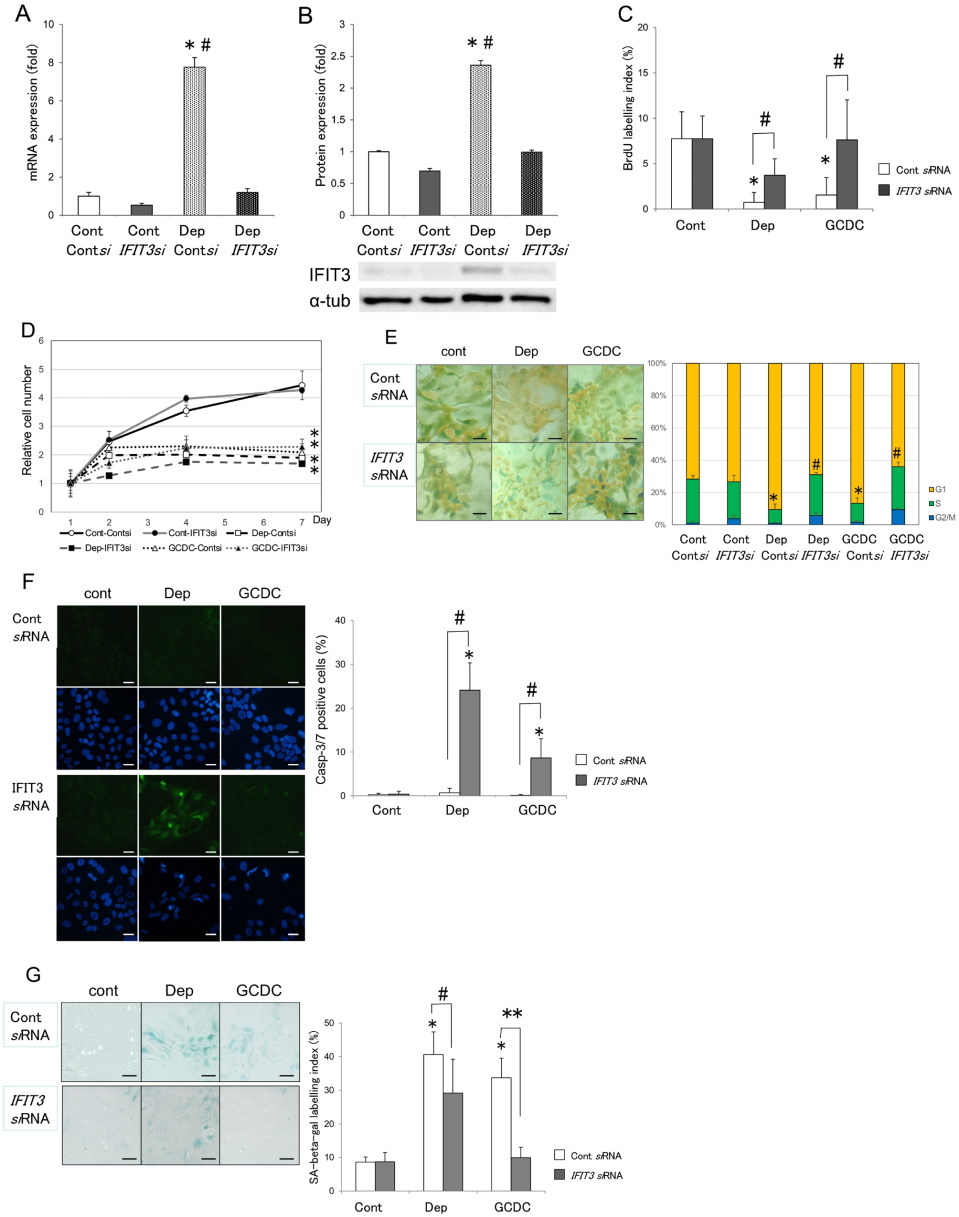
IFIT3 knockdown increased cell proliferation, apoptosis and decreased in cellular senescence in BECs treated with serum depletion and GCDC. (**A**, **B**) Effective knockdown of Ifit3. (**A**) The expression of Ifit3 mRNA in BECs treated with serum depletion and Ifit3 small interfering RNA (*si*RNA) or control *si*RNA for 1 day. Ifit3 mRNA was significantly increased in BECs treated with serum depletion (Dep) compared to the control (*p* < 0.01) and the increase was significantly suppressed by a treatment with Ifit3 *si*RNA compared to the control siRNA (*p* < 0.01). Data are expressed as the means ± SD. *p < 0.01 vs. control + Cont *si*RNA, ^#^*p* < 0.01 compared to Dep + Ifit3 *si*RNA. n = 3 for each group. (**B**) The protein level expression of IFIT3 assessed by immunoblotting in BECs treated with serum depletion and Ifit3 *si*RNA or control *si*RNA for 4 days. The protein level expression of IFIT3 was significantly increased in BECs treated with serum depletion compared to the control (*p* < 0.05) and the increase was significantly suppressed by a treatment with Ifit3 *si*RNA compared to the control siRNA (*p* < 0.05). *p < 0.05 vs. control + Cont *si*RNA; ^#^*p* < 0.05 compared to Dep + Ifit3 *si*RNA, n = 3 for each group. (**C**) Cell proliferation was increased by a treatment with Ifit3 *si*RNA. Cell proliferation activity was detected by BrdU assay. Cell proliferation activities of BECs after the induction of cellular senescence (serum depletion and GCDC) for 4 days with a treatment with Ifit3 *si*RNA or control *si*RNA. BrdU-LI is significantly lower in BECs treated with serum depletion or GCDC compared to the control (*p* < 0.01) and significantly higher in BECs treated with Ifit3 *si*RNA. The data are expressed as the mean ± SD. *p < 0.05 vs. Cont *si*RNA; ^#^*p* < 0.05. n = 5 for each group. (Con-Con*si*, 7.75 ± 2.95; Con-Ifit3*si*, 7.75 ± 2.50; Dep-Cont*si*, 0.72 ± 1.10; Dep-Ifit3*si*, 3.71 ± 1.84; GCDC-Cont*si*, 1.53 ± 1.94; Dep-Ifit3*si*, 7.60 ± 3.42). (**D**) Cell growth curve assessed by cell number was not changed by a treatment with Ifit3 *si*RNA. Cell number was assessed by WST assay after the induction of cellular senescence with serum deprivation (Dep) or GCDC (500 nM) with Ifit3 *si*RNA or control *si*RNA for 1, 2, 4 and 7 days. The data are expressed as the mean ± SD. *p < 0.01 vs. Con-Cont *si*RNA. n = 4 for each group. (**E**) G1/S arrest was resolved by a treatment with *IFIT3 si*RNA. Cell cycle was analyzed using Cell-Clock cell cycle assay on BECs by the treatment with serum depletion (Dep) or GCDC for 4 days with or without knockdown of *IFIT3* using *si*RNA. Cells become yellow in G1, green in S/G2 and blue in M phase. The data are expressed as the mean ± SD. *p < 0.01 vs. Con-Con*si*RNA; ^#^*p* < 0.05 between IFIT3 siRNA and Con*si*RNA in each condition. n = 4 for each group. (% S-phase: Con-Con*si*, 27.4 ± 2.2; Con-Ifit3*si*, 22.9 ± 4.0; Dep-Cont*si*, 8.6 ± 3.3; Dep-Ifit3*si*, 25.3 ± 1.4; GCDC-Cont*si*, 11.7 ± 3.3; Dep-Ifit3*si*, 26.6 ± 3.3). (**F**) Apoptosis was effectively induced in senescent BECs by a treatment with Ifit3*si*RNA. Apoptosis was assessed by detecting caspase-3/7 activity after the induction of cellular senescence with serum deprivation (Dep) or GCDC (500 nM) with Ifit3 *si*RNA or control *si*RNA for 4 days. Apoptotic cells showed caspase-3/7 activity with green fluorescence. The data are expressed as the mean ± SD. *p < 0.01 vs. Cont *si*RNA; ^#^*p* < 0.01. n = 5 for each group. (Con-Con*si*, 0.2 ± 0.3; Con-Ifit3*si*, 0.4 ± 0.64; Dep-Cont*si*, 0.7 ± 1.0; Dep-Ifit3*si*, 24.1 ± 6.2; GCDC-Cont*si*, 0.1 ± 0.2; Dep-Ifit3*si*, 8.7 ± 4.4). Scales are 10 μm. (**G**) Cellular senescence was effectively decreased in BECs with the induction of cellular senescence (serum depletion and GCDC) with a treatment with Ifit3 *si*RNA or control *si*RNA for 4 days. Cellular senescence was assessed by senescence-associated β-galactosidase activity (SA-β-gal) after treatment with serum deprivation (Dep) or GCDC (500 nM) and Ifit3 *si*RNA or control *si*RNA for 4 days. Percentage of cells positive for SA-β-gal was significantly increased in cells treated with Dep (SA-β-gal labeling index, 40.6 ± 6.8) or GCDC (33.7 ± 5.8). Treatment with Ifit3 *si*RNA significantly decreased cellular senescence in each condition (Dep + Ifit3 *si*RNA, 29.2 ± 6.0; GCDC + Ifit3 *si*RNA (9.9 ± 3.1). Data was expressed as mean ± SD. *p < 0.01 compared to control + Cont *si*RNA. ^#^*p* < 0.01, **p < 0.05. The data are expressed as the n = 5 for each group. Scales are 10 μm.

We examined the effect of *IFIT3* knockdown on BECs. An effective knockdown of *IFIT3* using *si*RNA was confirmed in mRNA and protein levels (Fig. 2A,B).

##### IFIT3 knockdown increased cell proliferation activity

Cell proliferation activity detected by BrdU assay after the induction of cellular senescence (serum depletion and GCDC) for 4 days with or without knockdown of *IFIT3* using *si*RNA. BrdU-labelling index was significantly low in BECs treated with serum depletion and GCDC for 4 days (*p* < 0.05) (Fig. 2C). The knockdown of *IFIT3* significantly increased cell proliferation activity in BECs treated with serum depletion and GCDC (*p* < 0.05) (Fig. 2C).

##### IFIT3 knockdown did not change the growth curve assessed by cell number

Cell growth was significantly arrested in BECs by the treatment with serum depletion or GCDC (*p* < 0.01). *IFIT3* knockdown did not change the growth curve in each condition in BECs (Fig. 2D). The increased proliferation and apoptosis may result in no change in the growth curve by treatments with serum depletion and GCDC.

##### IFIT3 knockdown resolved G1/S arrest

Cell cycle was analyzed using Cell-Clock cell cycle assay on BECs by the treatment with serum depletion or GCDC for 4 days with or without knockdown of *IFIT3* using *si*RNA. G1/S arrest was induced by the treatment with serum depletion or GCDC (*p* < 0.05) (Fig. 2E). G1/S arrest was significantly resolved by *IFIT3* knockdown in BECs treated with serum depletion or GCDC (*p* < 0.01) (Fig. 2E).

##### IFIT3 knockdown increased apoptosis

Caspase-3/7 activity was detected for assessment of apoptosis at 4 days after the induction of cellular senescence with or without knockdown of *IFIT3* using *si*RNA. Caspase-3/7 activity with green fluorescence was detected in apoptotic cells (Fig. 2F). Apoptotic cells were significantly increased in senescent BECs with knockdown of *IFIT3* (*p* < 0.01) (Fig. 2F).

##### IFIT3 knockdown decreased cellular senescence

Cellular senescence was assessed by the activity of SA-β-gal after treatment with serum deprivation or GCDC (500 nM) for 4 days with or without knockdown of *IFIT3* using *si*RNA (Fig. 2G). SA-β-gal labelling index, a marker of cellular senescence, was significantly increased by treatment with serum depletion or GCDC. Cellular senescence was significantly decreased by knockdown of *IFIT3* using *si*RNA in BECs treated with serum depletion or GCDC for induction of senescence (*p* < 0.01) (Fig. 2G).

### Human study

#### Increased expression of IFIT3 in senescent BECs in damaged small bile ducts in PBC

**Figure 3:**
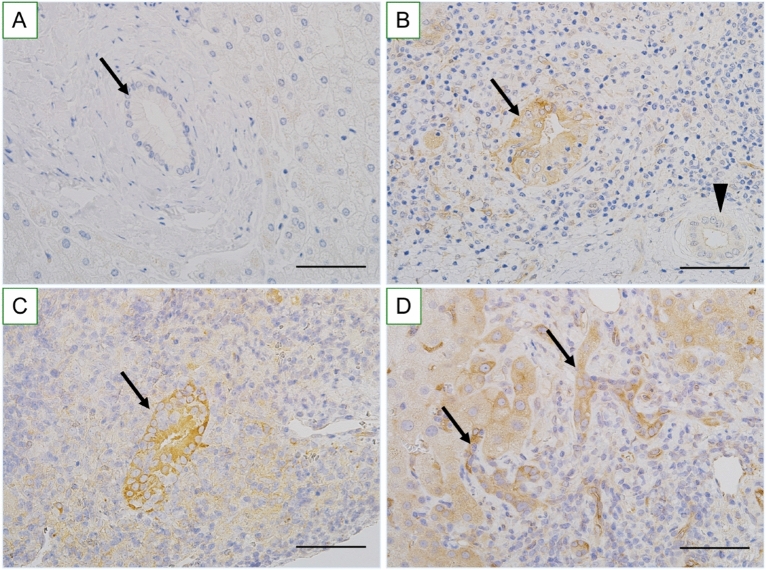
Increased expression of IFIT3 in small bile ducts and bile ductular cells in PBC and control livers. (**A**) IFIT3 is not expressed in small bile duct (arrow) in normal liver. (**B**) IFIT3 is expressed in biliary epithelial cells (BECs) involved in chronic nonsuppurative destructive cholangitis (arrow) in PBC. Arrowhead indicates a normal-looking small bile duct without IFIT3 expression. (**C**) Another example of IFIT3 expression in chronic nonsuppurative destructive cholangitis (arrow) in PBC. (**D**) IFIT3 is expressed in the cytoplasm of bile ductular cells in ductular reactions in PBC. Immunostaining for IFIT3. Original magnification, × 400. Scales are 50 μm.

IFIT3 was expressed in the cytoplasm in BECs, when present (Fig. 3A–D). The expression of IFIT3 was significantly increased in BECs in small bile ducts involved in cholangitis in PBC (Fig. 3B,C). Table 2 is a summary of the extent of IFIT3 expression in small bile ducts in PBC and control livers. The expression of IFIT3 was significantly more extent in small bile ducts in PBC, compared with control livers (*p* < 0.01). The expression of IFIT3 was significantly correlated with cholangitis activity in PBC (*p* < 0.01). Similar tendency was confirmed in another cohort of 15 patients with PBC (data not shown). The increased expression of IFIT3 in small bile duct was inversely correlated to inadequate response to UDCA in PBC (*p* < 0.05).

**Table 2:** Frequency of the expression of Interferon-induced protein with tetratricopeptide repeats 3 (IFIT3) in small bile ducts in primary biliary cholangitis and control livers.

Diseases	Number of patients	IFIT3 expression n (%)	[1+, 2+, 3+]
PBC	70	54 (77.1%)^a–d^	[6, 19, 29]
PBC, st1/2; CA3	33	28 (84.8%)	[2, 9, 17]
PBC, st1/2; CA0-2	12	8 (66.7%)	[2, 3, 3]
PBC, st3/4; CA3	9	9 (100%)^e^	[0, 2, 7]
PBC, st3/4; CA0-2	16	9 (56.3%)	[2, 5, 2]
CVH	61	22 (36.1%)	[16, 6, 0]
CVH, st1/2	38	11 (28.9%)	[9, 2, 0]
CVH, st3/4	23	11 (47.8%)	[7, 4, 0]
PSC	12	5 (41.7%)	[7, 4, 0]
EBO livers	10	3 (30.0%)	[3, 0, 0]
Normal livers	18	2 (11.1%)	[1, 1, 0]

PBC, primary biliary cholangitis; CVH, chronic viral hepatitis; PSC, primary sclerosing cholangitis; EBO, extrahepatic biliary obstruction; a, *p* < 0.01 versus CVH; b, *p* < 0.05 versus PSC; c, *p* < 0.01 versus EBO; d, *p* < 0.05 versus Normal livers; e, *p* < 0.01 versus PBC, stage 3/4, CA0-2; n, number; n, number; [ ], number of cases showing 1 + (focal, positive cells are detected in one third or fewer portal tracts), and 2 + (moderate, positive cells are detected in small bile ducts of more than one third of portal tracts); 3 + (extensive, positive cells are detected in small bile ducts of more than two thirds of portal tracts).

#### Increased expression of IFIT3 in senescent bile ductular cells in ductular reaction in PBC

The expression of IFIT3 was significantly increased in bile ductular cells in ductular reaction in PBC (Fig. 3D). Table 3 is a summary of the extent of IFIT3 expression in bile ductular cells in PBC and control livers. The expression of IFIT3 was significantly more extent in bile ductular cells in PBC, compared with control livers (*p* < 0.01). Similar tendency was confirmed in another cohort of 15 patients with PBC (data not shown).

**Table 3:** Frequency of the expression of Interferon-induced protein with tetratricopeptide repeats 3 (IFIT3) in bile ductules in primary biliary cholangitis and control livers.

Diseases	Number of patients	IFIT3 expression n (%)	[1+, 2+, 3+]
PBC	70	57 (81.4%)^a-c^	[20, 28, 9]
PBC, st1/2; CA3	33	27 (81.8%)	[9, 15, 3]
PBC, st1/2; CA0-2	12	10 (83.3%)	[6, 4, 0]
PBC, st3/4; CA3	9	8 (88.9%)^d^	[0, 4, 4]
PBC, st3/4; CA0-2	16	11 (68.8%)	[5, 2, 2]
CVH	61	22 (36.1%)	[16, 6, 0]
CVH, st1/2	38	9 (23.7%)	[7, 2, 0]
CVH, st3/4	23	13 (56.5%) ^e^	[9, 4, 0]
PSC	12	5 (41.7%)	[2, 3, 1]
EBO livers	69	3 (30.0%)	[3, 0, 0]
Normal livers	32	1 (5.6%)	[1, 0, 0]

PBC, primary biliary cholangitis; CVH, chronic viral hepatitis; PSC, primary sclerosing cholangitis; EBO, extrahepatic biliary obstruction; a, *p* < 0.01 versus CVH; b, *p* < 0.01 versus EBO; c, *p* < 0.01 versus Normal livers; d, *p* < 0.05 versus stage 3/4, CA0-2; e, *p* < 0.01 versus CVH, stage 1/2; n, number; [ ], number of cases showing 1 + (focal, positive cells are detected in one third or fewer portal tracts), and 2 + (moderate, positive cells are detected in bile ductules in more than one third of portal tracts), 3 + (extensive, positive cells are detected in bile ductules in more than two thirds of portal tracts).

#### Increased expression of IFIT3 in senescent BECs in damaged small bile ducts and bile ductules in PBC

**Figure 4:**
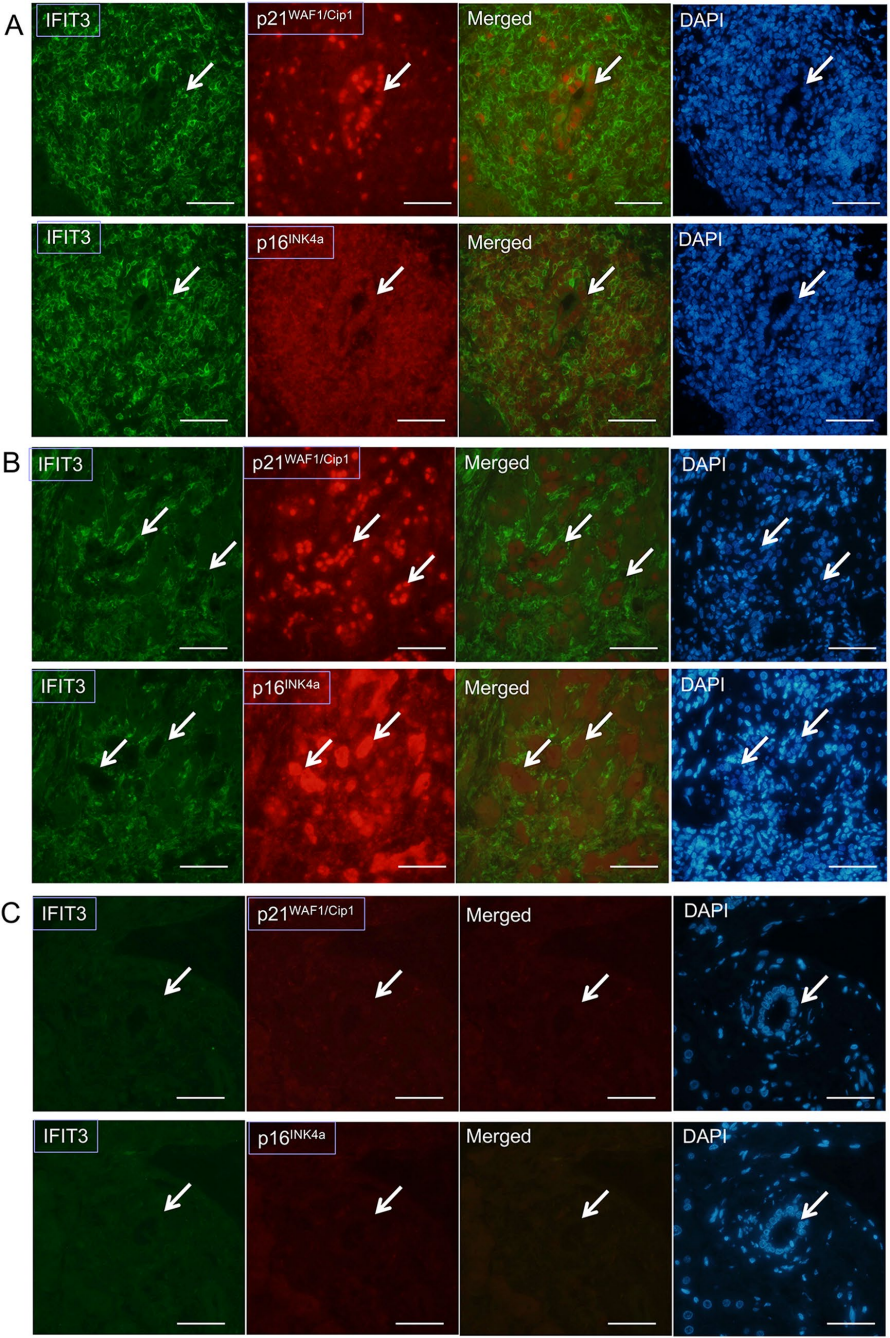
Association of IFIT3 expression with senescent markers p16^INK4a^ in small bile ducts in primary biliary cholangitis (PBC). (**A**) Increased expression of p21^WAF1/Cip1^ was seen in the nuclei of BECs in a damaged small bile duct (arrow) showing increased expression of IFIT3 in PBC. Increased expression of p16^INK4a^ was seen in the nuclei of BECs in a damaged small bile duct (arrow) showing increased expression of IFIT3 in PBC. Double immunostaining for IFIT3 (green) and p21^WAF1/Cip1^ or p16^INK4a^ (red). Original magnification, × 400. Scales are 50 μm. (**B**) Increased expression of p21^WAF1/Cip1^ was seen in the nuclei of BECs in ductular cells in ductular reaction (arrows) showing increased expression of IFIT3 in PBC. Increased expression of p16^INK4a^ was seen in the nuclei and cytoplasm of BECs in ductular cells in ductular reaction (arrows) showing increased expression of IFIT3 in PBC. Double immunostaining for IFIT3 (green) and p21^WAF1/Cip1^ or p16^INK4a^ (red). Original magnification, × 400. Scales are 50 μm. (**C**) IFIT3, p21^WAF1/Cip1^ and p16^INK4a^ were not expressed in BECs in a small bile duct (arrow) in control normal liver. Double immunostaining for IFIT3 (green) and p21^WAF1/Cip1^ or p16^INK4a^ (red). Original magnification, × 400. Scales are 50 μm.

Double immunostaining revealed that the expression of IFIT3 was frequently increased in BECs in the senescent small bile ducts showing expression of p21^WAF1/Cip1^ or p16^INK4a^ in PBC (Fig. 4A). The increased expression of IFIT3 was also seen in senescent BECs with expression of p16^INK4a^ and p21^WAF1/Cip1^ in ductular reactions in PBC (Fig. 4B). Since intracytoplasmic localization of p21^WAF1/Cip1^ (nucleus) or p16^INK4a^ (nucleus and cytoplasm) are different from IFIT3 (cytoplasm), the co-localization was not indicated by yellow color in the merged images (Fig. 4A,B). However, the expression was seen in same BECs. Figure 4C shows a view of double fluorescent stain in control normal liver.

## Discussion

The findings are summarized as follows; (1) Senescent BECs induced by both serum depletion or a treatment with GCDC for 7 days showed common upregulation of 1841 genes (fold change > 2), which included *CCL2*, *IFIT3, CPQ and* a number of chemokines and cytokines; (2) Cell proliferation and apoptosis were significantly increased (*p* < 0.01) and cellular senescence was significantly decreased (*p* < 0.01) by knockdown of *IFIT3* in BECs treated with serum depletion or GCDC; (3) Small bile ducts showing cholangitis and in ductular cells in ductular reactions expressed IFIT3 in significantly higher level in PBC, compared to control livers; (4) Cholangitis activity was significantly correlated with the expression of IFIT3 in small bile ducts in PBC (*p* < 0.01); (5) Senescent BECs showing the expression of p16^INK4a^ and p21^WAF1/Cip1^ in bile duct lesions in PBC expressed IFIT3 in high level; (6) Inadequate response to UDCA was inversely correlated to the increased expression of IFIT3 in small bile duct in PBC (*p* < 0.05).

In this study, we examined comprehensive profiles of senescent BECs relating to cholangiopathies. The results showed that the expression of various chemokines and cytokines are increased as SASPs in agreement with our previous studies^8,15,16^. For example, CCL2, which we reported an increased expression and possible roles in the pathogenesis of PBC^8,16^, is included in the top 5 commonly-upregulated genes in senescent BECs in this study. It is now well known that senescent BECs express various chemokines and cytokines as SASPs in PBC and PSC, which may modulate inflammatory cell infiltration and fibrosis in cholangiopathies^8,15,16,25^. Altered expression of various inflammatory factors is also known in senescent cells^9,13,14,26^.

Among these inflammatory factors, we put focus on one of the top5 commonly-upregulated genes; IFIT3^17–19^ in senescent BECs in this study. In vitro study confirmed the increased expression of IFIT3 in mRNA and protein levels in this study, confirming the data of mRNA array study. In the present study, we found for the first time the increased expression of IFIT3 relating to cellular senescence in BECs in PBC. Increased expression of IFIT3 and its association with pathophysiology were reported in several diseases such as SLE^27^, RA^28^ and degenerative annulus fibrosus^29^. IFIT3 is an IFN-induced protein and upregulates cGMP-AMP synthase (cGAS)-signal via stimulator of IFN genes protein (STING) pathway^27,29^. cGAS-STING pathway is known to play roles in the process of cellular senescence such as the expression of SASPs^30^. Taken together, IFIT3 may also contribute to the overactivation of cGAS/STING signaling pathway in BECs and may participate in the pathophysiology of PBC. Further studies are mandatory to confirm an involvement of IFIT3 on signal transduction pathways.

Accumulating evidences suggest that IFN pathways may participate in the pathogenesis of PBC^20–24^, however, there has been no report regarding IFIT3 in PBC to our knowledge. Recent integrated analysis using GWAS, and mRNA microarray data sets predicted that IFNG and CD40L are the central upstream regulators in both disease susceptibility and activity of PBC^24^. The interplay of types I and II IFN is also implicated as a cause of human PBC^23^ and a murine model of autoimmune cholangitis^22^. A previous paper implicated type I IFN signaling as a necessary component of the sex bias in the murine model of autoimmune cholangitis with chronic IFN–γ stimulating^22^. The previous paper also suggested that drugs that target the type I IFN signaling pathway would have potential benefit in the earlier stages of PBC^22^. IFN‐γ but not IFN‐β, TGF‐β or TNFα was found to up‐regulate STING expression in keratinocytes^31,32^. Taken together, the present study suggests that IFIT3 may be a key molecule, which participates in the crossroads of IFN signaling, cGAS-STING pathway and cellular senescence in PBC.

In the present study, cellular senescence was decreased, and cell proliferation was increased by a knockdown of *IFIT3* in BECs. Furthermore, G1/S arrest was resolved by a knockdown of *IFIT3* in BECs treated with serum depletion or GCDC. A previous study reported significant accumulation of cells at G1/S transition in human monocytic cells with ectopic expression of IFIT3^18^. IFIT3 has been shown to have anti-proliferative activity by enhancing the expression of cell cycle negative regulators such as p27 and p21 by downregulating c-Myc^18^. The findings in the present study agree with previous study, suggesting that IFIT3 may contribute to induce cellular senescence.

A knockdown of IFIT3 in BECs treated with serum depletion or GCDC significantly increased apoptosis in the present study. Previous study suggested that upregulation of IFIT3 plays a protective role in lung epithelial cells in dengue virus infection by an inhibition of apoptosis^33^. IFIT3 knockdown induced apoptosis and suggested that the apoptotic effects of IFIT2 could be negatively regulated by IFIT3^34^. The findings in the present study agree these previous studies and confirmed anti-apoptotic roles of IFIT3 overexpression. It is well known that senescent cells are resistant to apoptosis^26,35^. “Senescent cells and anti-apoptotic pathways” (SCAPs) including Bcl-xL are thought to have responsibility for this resistant mechanism^26,35^. Since IFIT3 is upregulated in senescent cells and inhibits apoptosis^33,34^, increased expression of IFIT3 in bile duct lesions in senescent BECs in PBC may contribute to the anti-apoptosis as one of SCAPs. Taken together, IFIT3 may serve as a novel therapeutic target for clearance of senescent cells and for blocking the production of type I IFN and other proinflammatory cytokines by the cGAS/STING signaling pathway in PBC.

In conclusion, senescent BECs showed increased expression of various genes related to immunity and inflammation including SASPs. The increased expression of IFIT3 in BECs may be involved in the pathogenesis of PBC and could be a therapeutic target in PBC.

## Methods

### Culture study

#### Cell culture and treatments

Mouse intrahepatic BECs were isolated from 8-week-old female BALB/c mice and were purified and cultured as described previously^36,37^. All methods were carried out in accordance with relevant guidelines and regulations. The cell density of the cells was less than 80% during experiments. Cellular senescence was induced in BECs cultured in vitro incubation in serum depleted media or treatment with media containing 200 μM GCDC for 4–7 days, as described previously^37–39^. A treatment with GCDC causes cellular senescence via the induction of endoplasmic reticulum stress and dysregulated autophagy, as demonstrated in previous studies^15,37–39^.

#### RNA extraction and cDNA microarray

Total RNA was extracted from the cells with a QIAGEN RNeasy Mini kit (QIAGEN, Hilden, Germany), as described previously^37^ according to the manufacturer’s protocol. Genome-wide expression profiling was performed using a 3D-Gene scanner with 3D-Gene Oligo chip 24 k (Toray Industries, Inc., 23,522 distinct genes) and the supplier's protocol. Hybridization signals were scanned 3D-Gene Scanner (Toray Industries) and processed by 3D-Gene Extraction software (Toray Industries). The raw data of each spot was normalized by subtraction with a mean intensity of the background signal determined by all blank spots' signal intensities of 95% confidence intervals. The raw data intensities greater than 2 standard deviations (SD) of the background signal intensity were considered to be valid. cDNA microarray was performed using pooled RNA samples from 2 independent experiments for each condition. Second cDNA microarray was performed using different sets of samples for each condition and major up-regulated genes including IFIT3 were confirmed. Data analysis and functional analysis was performed by Gene Ontologies and KEGG pathway analysis. GSEA was performed according to the instructions.

#### Data deposition

Microarray data are deposited in the National Center for Biotechnology Information Gene Expression Omnibus database (accession number GSE168052).

#### Knockdown of Ifit3 by small interfering RNA (*si*RNA)

Validated *si*RNA for Ifit3 and negative control *si*RNA were purchased from Santa-Cruz biotech (Santa-Cruz, CA, USA) and QIAGEN, respectively. One day before transfection, BECs were plated in 35 mm-dishes (5 × 10^5^ cells), 96-well plate (1 × 10^4^ cells/well) or 12-well plate (5 × 10^4^ cells/well), and then the cells were transiently transfected with either Ifit3 or control *si*RNA (100 nM) using Lipofectamine 3000 (Invitrogen, Carlsbad, CA), as described previously^37^ according to the manufacturer’s protocol.

#### Real-time quantitative reverse transcriptase-polymerase chain reaction

After cDNA was synthesized, quantitative real-time PCR was performed to measure Ifit3 and β-actin mRNA, as described previously^37^ according to a standard protocol using the SYBR Green PCR Master Mix (Toyobo, Tokyo, Japan). Forward and reverse primers 5′-GAGTGCTGCTTATGGGGAGA and 5′-AGAGCAGTTTGTCAGCAATCC, respectively, were used for Ifit3 and 5′-CCACCGATCCACACAGAGTA and 5′-GGCTCCTAGCACCATGAAGA for β-actin as an internal control. Each experiment was performed twice in triplicate, and the mean was calculated for each of the experiments.

##### Immunoblotting

The cell lysate samples (10 μg) were resolved by SDS-PAGE and transferred to a nitrocellulose membrane as described previously^37^. After transfer, the membranes were processed for immunoblotting as described previously^37^. The primary antibodies used were shown in supplementary table S2. Densitometry of the resulting bands was performed using Image-J software and normalized to the loading control.

##### Assay for cell proliferation

Cell proliferation activity was assessed on day 4 after treatment by using a 5-bromo-2′-deoxy-uridine (BrdU) Labeling and Detection Kit (Roche, Nonenwald, Germany), according to manufactures’ protocol. The nuclei were simultaneously stained with DAPI. At least 1 × 10^3^ total cells were checked and counted to assess the BrdU-labeling index with a conventional fluorescence microscope (Olympus).

##### Assay for cell number

BECs were seeded into 96-well microplates (1 × 10^4^ cells/well) and incubated in a final volume of 100 μl medium. The cell number was assessed on days 1, 2, 4 and 7 after treatment using a Cell Proliferation Reagent WST-1 (Roche, Basel, Switzerland) according to manufacturer’s recommendation.

##### Assay for cell cycle

The cell cycle alteration was detected on day 4 after treatment by a Cell‐Clock Assay Kit (Biocolor, Northern Ireland, UK), according to manufacturer’s protocol. Images of the cells were acquired using a conventional microscope (Olympus) and analyzed using Image J software.

##### Assay for apoptosis

The apoptotic cells in each condition were assessed after the induction of cellular senescence and a treatment with senolytic reagents by using CellEvent Caspase-3/7 Green Detection Reagent (Life Technologies, Carlsbad, CA) as described previously^39^ according to manufacturer’s protocol. The nuclei were simultaneously stained with DAPI. At least 1 × 10^3^ total cells were checked and counted to assess the percentage of apoptotic cells showing Caspase-3/7 activity with a conventional fluorescence microscope.

##### Assay for cellular senescence

The activity of senescence-associated β-galactosidase **(**SA-β-gal) was detected after the induction of cellular senescence and a treatment with senolytic reagents by using the senescence detection kit (Bio Vision, Mountain View, CA) according to manufacturer’s protocol^40^. The proportion of senescent cells was assessed by counting SA-β-gal-positive cells in at least 1 × 10^3^ total cells.

### Human study

#### Classification of intrahepatic biliary tree

The intrahepatic biliary tree is classified into intrahepatic large and small bile ducts (septal and interlobular bile ducts) by their size and distributions in the portal tracts^41^. Bile ductules, which are characterized by tubular or glandular structures with a poorly defined lumen and located at the periphery of the portal tracts^41,42^, are not included in the small bile ducts and evaluated separately.

#### Liver tissue preparation

A total of 171 liver tissue specimens (all were biopsied or surgically resected) were collected from the liver disease file of our laboratory and affiliated hospitals. All methods were carried out in accordance with the Declaration of Helsinki, relevant guidelines and regulations. The Ethics Committee of Kanazawa University approved this study. The liver specimens in this study were 70 PBC, 61 chronic viral hepatitis (CVH), 12 PSC, 10 extrahepatic biliary obstruction (EBO) and 18 “histologically normal” livers. All PBC specimens were from patients fulfilling the clinical, serological and histological characteristics consistent with the diagnosis of PBC^2^ and histologically classified according to Nakanuma classification^43^. Table 4 is a summary of the clinicopathological features of the PBC patients included in the present study.

**Table 4:** Summary of clinicopathological features of patients with primary biliary cholangitis examined.

*Number of patients*	*70*
Age (mean ± SD; range)	58.9 ± 10.7(31–87)
Sex (male/female)	9/61
Anti-mitochondrial antibody	61 (87.1%)
Anti-nuclear antibody	44 (62.9%)
Other autoimmune disease	18 (25.7%)
Family history	5 (7.1%)
UDCA therapy	17 (24.3%)
UDCA inadequate responder	17 (24.3%)
Scheuer stage (stages1,2/3,4)	45/25
Nakanuma stage (stages 1,2/3,4)	45/25
Cholangitis activity (CA)(CA0-2/CA3)	28/42
Hepatitis activity (HA) (HA0,1/HA2,3)	48/22

*UDCA* ursodeoxycholic acid.

Seventeen PBC livers were after UDCA therapy and 17 were UDCA non-responders. Thirty-eight and 23 CVH patients were regarded as F0-2 and as F3, 4, respectively^44^. Ten and 61 of CVH cases were serologically positive for hepatitis B surface antigen and anti-hepatitis C viral antibody, respectively. Causes of EBO were obstruction of the bile duct at the extrahepatic bile ducts or the hepatic hilum due to stone or carcinoma, and the duration of jaundice was less than 1 month. “Histologically normal” livers were obtained from surgically resected livers for metastatic liver tumor or traumatic hepatic rupture. Normal liver tissues were obtained from an area apart from the tumor and carcinoma tissues were not evaluated.

Liver tissue samples were fixed in 10% neutral-buffered formalin and embedded in paraffin. More than twenty serial sections, 4 μm-thick, were cut from each block. Several sections were processed routinely for histologic study, and the remainder was processed for the subsequent immunohistochemistry.

##### Immunohistochemistry

The expression of IFIT3 and senescence-related markers p16^INK4a^, p21^WAF1/Cip1^ were examined, as described previously^12^. The primary antibodies used are shown in supplementary table S3. A similar dilution of the control mouse or rabbit Immunoglobulin G (Dako) was applied instead of the primary antibody as a negative control. Positive and negative controls were routinely included. Histological analysis was performed in a blinded manner. BECs in small bile ducts and bile ductules were separately evaluated.

#### Extent of IFIT3 expression in small bile ducts and bile ductules

The extent of expression was evaluated as follows: 1+, focal, positive cells are detected in one third or fewer portal tracts; 2+, moderate, positive cells are detected in small bile ducts of more than one third of portal tracts; 3+, extensive, positive cells are detected in small bile ducts of more than two thirds of portal tracts.

##### Double immunofluorescence

Double immunofluorescence for IFIT3 with senescent markers (p16^INK4a^ and p21^WAF1/Cip1^) was also performed. In brief, either of p16^INK4a^ or p21^WAF1/Cip1^ was detected using Vector Red Alkaline Phosphatase Substrate Kit (Vector Lab, Burlingame, CA), followed by second staining for Ifit3 using Alexa-488-labeled anti-rabbit IgG. The sections were counterstained with DAPI and evaluated under a conventional fluorescence microscope.

### Statistical analysis

Statistical analysis of differences was performed using the Kruskal–Wallis test with Dunn’s posttest. When the number of groups is 2, statistical analysis of difference was performed using the Mann–Whitney test. The Chi-square test or Fisher’s exact test was used to analyze categorical data. The correlation coefficient of 2 factors was evaluated using Spearman’s rank correlation test. When the *P* value was less than 0.05, the difference was regarded as significant. All analyses were performed using the GraphPad Prism software (GraphPad Software, San Diego, CA, USA).

## Supplementary Information

Supplementary Information.
