# Supplementary material for: Interferon-induced protein with tetratricopeptide repeats 3 may be a key factor in primary biliary cholangitis
Source: Sci Rep. 2021 Jun 1;11:11413. doi: 10.1038/s41598-021-91016-6 (PMC8169865; doi:10.1038/s41598-021-91016-6)
Supplement: Supplementary file 1 — Supplementary Information. [file 41598_2021_91016_MOESM1_ESM.pdf]

Supplementary information

Interferon-induced protein with tetratricopeptide repeats 3 may be a key factor in primary biliary cholangitis

Motoko Sasaki, MD, PhD[1)], Yasunori Sato MD, PhD [1)] and Yasuni Nakanuma MD, PhD [2)]
1) Department of Human Pathology, Kanazawa University Graduate School of Medical Sciences, Kanazawa, 920-8640, Japan
2) Department of Pathology, Fukui Saiseikai Hospital, Fukui 918-8503, Japan

Supplementary table S1, Related to table 1. Microarray expression analysis derived from senescent BECs induced by GCDC treatment and serum depletion. The fold change in gene expressions in GCDC treatment ant serum depletion samples relative to control was calculated. The genes that were significantly different using a 8-fold cut-off are listed. Global normalization value that is more than 100 is indicated in yellow.

Up-regulated transcripts in senescent BECs									global normalization			Dep/Contol		GCDC/Control	
Name	ID	RefSeq_id	Enterz_ic	symbol	description	GO_biologi	GO_molecu	GO_cellular	Control	Dep	GCDC	ratio	LOG2ratio	ratio	LOG2ratio
M200000049	M200000049	NM_011333.3	20296	Ccl2	chemokine (C-C m	GO:0001525	GO:0001664	GO:0005576	15	369	151	24.73	4.63	10.12	3.34
M400007854	M400007854	NM_010501.2	15959	Ifit3	interferon-induced	GO:0002376	GO:0003723	GO:0005737	7	306	87	42.50	5.41	12.08	3.59
M200003355	M200003355	NM_018755.2	54381	Cpq	carboxypeptidase	GO:0006508	GO:0004180	GO:0005576	4	44	40	10.68	3.42	9.72	3.28
M200003674	M200003674	NM_019738.1	56312	Nupr1	nuclear protein tra	GO:0002526	GO:0003677	GO:0005634	939	12877	8648	13.72	3.78	9.21	3.20
M200013916	M200013916	NM_019686.5	56506	Cib2	calcium and integ	GO:0007204	GO:0000287	GO:0001750	14	118	139	8.24	3.04	9.70	3.28
M400004927	M400004927	NM_001289655	225288	Fhod3	formin homology 2	GO:0007015	GO:0003674	GO:0005737	4	54	43	12.56	3.65	10.01	3.32
M200001306	M200001306	NM_207650.4	13527	Dtna	dystrobrevin alpha	-	GO:0005515	GO:0005737	3	89	60	28.92	4.85	19.61	4.29
M300000499	M300000499	NM_010360.2	14866	Gstm5	glutathione S-tran	GO:0006749	GO:0004364	GO:0005634	117	1672	954	14.29	3.84	8.15	3.03
M400013256	M400013256	XR_880180.1	69884	2010300F	RIKEN cDNA 201	GO:0008150	GO:0003674	GO:0005575	3	55	48	17.18	4.10	14.98	3.90
M300022199	M300022199	NM_001168578	66684	Tceal8	transcription elong	GO:0006351	GO:0050699	GO:0005634	35	861	879	24.76	4.63	25.27	4.66
M300017127	M300017127	NR_126154.1	104709	Pik3r6	phosphoinositide-	GO:0001525	GO:0046934	GO:0005737	41	987	347	24.32	4.60	8.55	3.10
M300005569	M300005569	NM_146125.2	228550	Itpka	inositol 1,4,5-trisp	GO:0006020	GO:0000166	GO:0043197	3	157	38	48.11	5.59	11.74	3.55
M200014447	M200014447	NM_145435.1	217212	Pyy	peptide YY	GO:0007218	GO:0001664	GO:0005576	1	33	14	27.12	4.76	11.51	3.52
M200002417	M200002417	NM_207105.3	14961	H2-Ab1	histocompatibility	GO:0002344	GO:0005515	GO:0005622	2	23	52	11.30	3.50	25.98	4.70
M300016369	M300016369	NM_018859.2	56043	Akr1e1	aldo-keto reducta	GO:0055114	GO:0016491	GO:0005737	2	17	17	8.08	3.01	8.27	3.05
M400003646	M400003646	NM_011469.3	20756	Sprr2b	small proline-rich p	GO:0008544	GO:0005198	GO:0001533	255	3288	2269	12.89	3.69	8.89	3.15
M200009840	M200009840	NM_022435.2	64406	Sp5	trans-acting trans	GO:0006351	GO:0000977	GO:0005634	5	163	42	32.18	5.01	8.26	3.05
M400012446	M400012446	NM_177915.4	209268	Igsf1	immunoglobulin su	GO:0006355	GO:0004872	GO:0005576	1	11	10	10.30	3.37	8.61	3.11

Supplementary table S2. GSEA. Supplementary table S2. Gene Set Enrichment Analysis on top 500 upregulated genes derived from senescent BECs induced by serum depletion and GCDC treatment. Gene sets including IFIT3 are marked. Bold indicates commonly detected gene sets in both conditions.

Upregulated Gene-Sets in senescenct BECs induced by serum depletion	No. of Enriched Genes	NOM p-val	IFIT3	Upregulated Gene-Sets in senescenct BECs induced GCDC	No. of Enriched Genes	NOM p-val	IFIT3
GO_MOLECULAR_TRANSDUCER_ACTIVITY	21	0.0043		GO_ION_HOMEOSTASIS	25	0.0022	
GO_RESPONSE_TO_HORMONE	18	0.0033		GO_G_PROTEIN_COUPLED_RECEPTOR_SIGNALING_PATHWAY	22	0.0033	
GO_POSITIVE_REGULATION_OF_CELL_POPULATION_PROLIFERATION	22	0.0396		GO_METAL_ION_HOMEOSTASIS	24	0.0055	
GO_RESPONSE_TO_WOUNDING	24	0.0482		GO_CELLULAR_ION_HOMEOSTASIS	21	0.0091	
GO_HUMORAL_IMMUNE_RESPONSE	15	0.0169	*	GO_CHEMICAL_HOMEOSTASIS	31	0.0042	
GO_EPITHELIAL_CELL_DIFFERENTIATION	17	0.0385		GO_DEFENSE_RESPONSE_TO_OTHER_ORGANISM	30	0.0119	*
GO_RESPONSE_TO_NITROGEN_COMPOUND	27	0.0611		GO_DIVALENT_INORGANIC_CATION_HOMEOSTASIS	15	0.0211	
GO_NEGATIVE_REGULATION_OF_DEVELOPMENTAL_PROCESS	29	0.0368		GO_INNATE_IMMUNE_RESPONSE	26	0.0174	*
GO_NEGATIVE_REGULATION_OF_CELL_DIFFERENTIATION	24	0.0494		GO_CELLULAR_HOMEOSTASIS	29	0.0282	
GO_EPITHELIAL_CELL_PROLIFERATION	18	0.0388		GO_DEFENSE_RESPONSE	55	0.0184	*
GO_WOUND_HEALING	21	0.0540		GO_RECEPTOR_REGULATOR_ACTIVITY	21	0.0366	
GO_RESPONSE_TO_ENDOGENOUS_STIMULUS	36	0.0153		GO_CYTOKINE_MEDIATED_SIGNALING_PATHWAY	24	0.0422	*
GO_INFLAMMATORY_RESPONSE	29	0.0735		GO_CELL_CELL_SIGNALING	32	0.0372	
GO_BIOLOGICAL_ADHESION	36	0.0746		GO_SYNAPSE	24	0.0509	
**GO_RESPONSE_TO_BIOTIC_STIMULUS**	44	0.0518	*	**GO_RESPONSE_TO_BIOTIC_STIMULUS**	42	0.0290	*
GO_EPITHELIUM_DEVELOPMENT	24	0.0577		GO_RESPONSE_TO_CYTOKINE	36	0.0432	*
GO_NEGATIVE_REGULATION_OF_MULTICELLULAR_ORGANISMAL_PROCE	40	0.0459		GO_ENZYME_REGULATOR_ACTIVITY	32	0.0535	
GO_REGULATION_OF_RESPONSE_TO_STRESS	37	0.0958		GO_AMIDE_BINDING	16	0.0633	
GO_CELL_CELL_ADHESION	20	0.0588		HP_CONSTITUTIONAL_SYMPTOM	19	0.0570	
GO_IDENTICAL_PROTEIN_BINDING	46	0.0153	*	GO_ENZYME_INHIBITOR_ACTIVITY	16	0.0611	

Supplementary table S3. Primary antibodies used in this study.

Primary antibody	*Type (clone)*	*Pre-treatment (FFPE)*	*dilution*	*Source*
Immunohistochemistry				
Ifit3	Rabbit poly	eARI –BA (121℃, 5min)	1:400	Proteintech, Rosemont, IL
Ifit3	Mouse mono (B-7)	eARI –BA (121℃, 5min)	1:200	Santa-Cruz, Santa-Cruz, CA
$p16^{INK4a}$	Mouse mono (JC8)	eARI –BA (121℃, 5min)	1:100	Neomarkers, Freemont, CA
$p21^{WAF1/Cip1}$	Mouse mono (70)	eARI –BA (121℃, 5min)	1:100	BD Transduction, San Jose, CA
Immunoblotting				
Ifit3	Rabbit poly		1:200	MyBiosource, San Diego, CA
α-tubulin	Mouse mono (TU-01)		1:500	Thermo Fisher, IL

Ifit3, interferon- induced protein with tetratricopeptide repeats 3, RT, room temperature; eARI, electronic antigen retrieval instrument (pascal, Dako); BA, 0.05M boric acid buffer (pH 8).

Supplementary information 4

Full blot of figure 1B and 2B

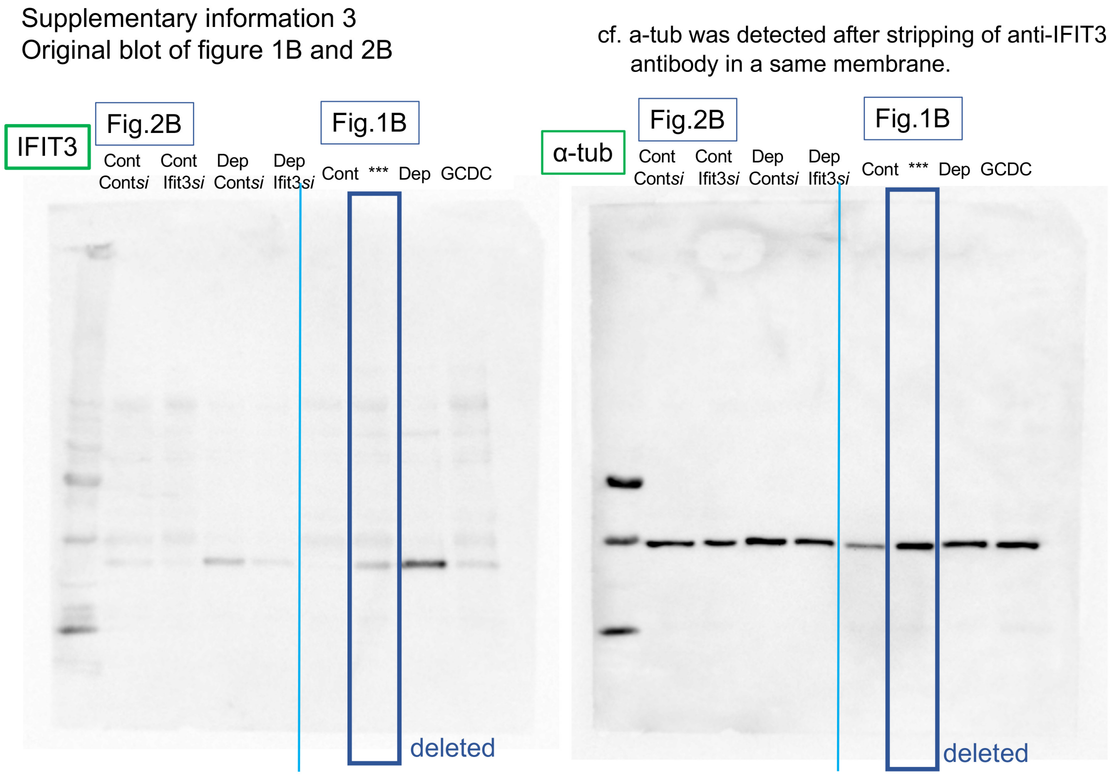

cf. α-tub was detected after stripping of anti-Ifit3 antibody in a same membrane.
